# BACA: bubble chArt to compare annotations

**DOI:** 10.1186/s12859-015-0477-4

**Published:** 2015-02-05

**Authors:** Vittorio Fortino, Harri Alenius, Dario Greco

**Affiliations:** Unit of Systems Toxicology, Finnish Institute of Occupational Health (FIOH), Topeliuksenkatu 41b, 00250 Helsinki, Finland; Nanosafety Centre, Finnish Institute of Occupational Health (FIOH), Topeliuksenkatu 41b, 00250 Helsinki, Finland

**Keywords:** Enrichment analysis, Visualize enrichment results, R package

## Abstract

**Background:**

DAVID is the most popular tool for interpreting large lists of gene/proteins classically produced in high-throughput experiments. However, the use of DAVID website becomes difficult when analyzing multiple gene lists, for it does not provide an adequate visualization tool to show/compare multiple enrichment results in a concise and informative manner.

**Result:**

We implemented a new R-based graphical tool, BACA (Bubble chArt to Compare Annotations), which uses the DAVID web service for cross-comparing enrichment analysis results derived from multiple large gene lists. BACA is implemented in R and is freely available at the CRAN repository (http://cran.r-project.org/web/packages/BACA/).

**Conclusion:**

The package BACA allows R users to combine multiple annotation charts into one output graph by passing DAVID website.

**Electronic supplementary material:**

The online version of this article (doi:10.1186/s12859-015-0477-4) contains supplementary material, which is available to authorized users.

## Background

High-throughput technologies, such as microarrays and RNA-sequencing, typically produce long lists of differentially expressed genes or transcripts, which are interpreted using functional annotation tools. One of the most used functional annotation program is DAVID [1,2]. The DAVID Bioinformatics Resources [1,2] at http://david.abcc.ncifcrf.gov provides an integrated biological knowledgebase and analytic tools to help users quickly find significantly represented biological themes (*e.g.* gene ontologies or pathways) in lists of pre-selected genes. DAVID functional annotation tool typically compiles biological terms enriched (overrepresented) in a list of up- or down-regulated genes, for instance from a transcriptomics experiment, in tabular format, which might be difficult to understand when comparing multiple experimental conditions (e.g. treatments, disease states, etc.). Several tools are available to visually compare the results from multiple enrichment analysis, such as GOBar [3], Go-Mapper [4], high-throughput GoMiner [5], the GOEAST [6] and REViGO [7]. These are specific tools that focus more on the integration than the visualization aspect.

Here we provide BACA, a novel R-based package to concisely visualize DAVID annotations across different experimental conditions. It makes use of the R package RDAVIDWebService [8] to query the DAVID knowledgebase and the advanced graphical functions provided by the R package ggplot2 (http://ggplot2.org) to build charts showing multiple enrichment analysis results across conditions.

## Implementation

**Figure 1:**
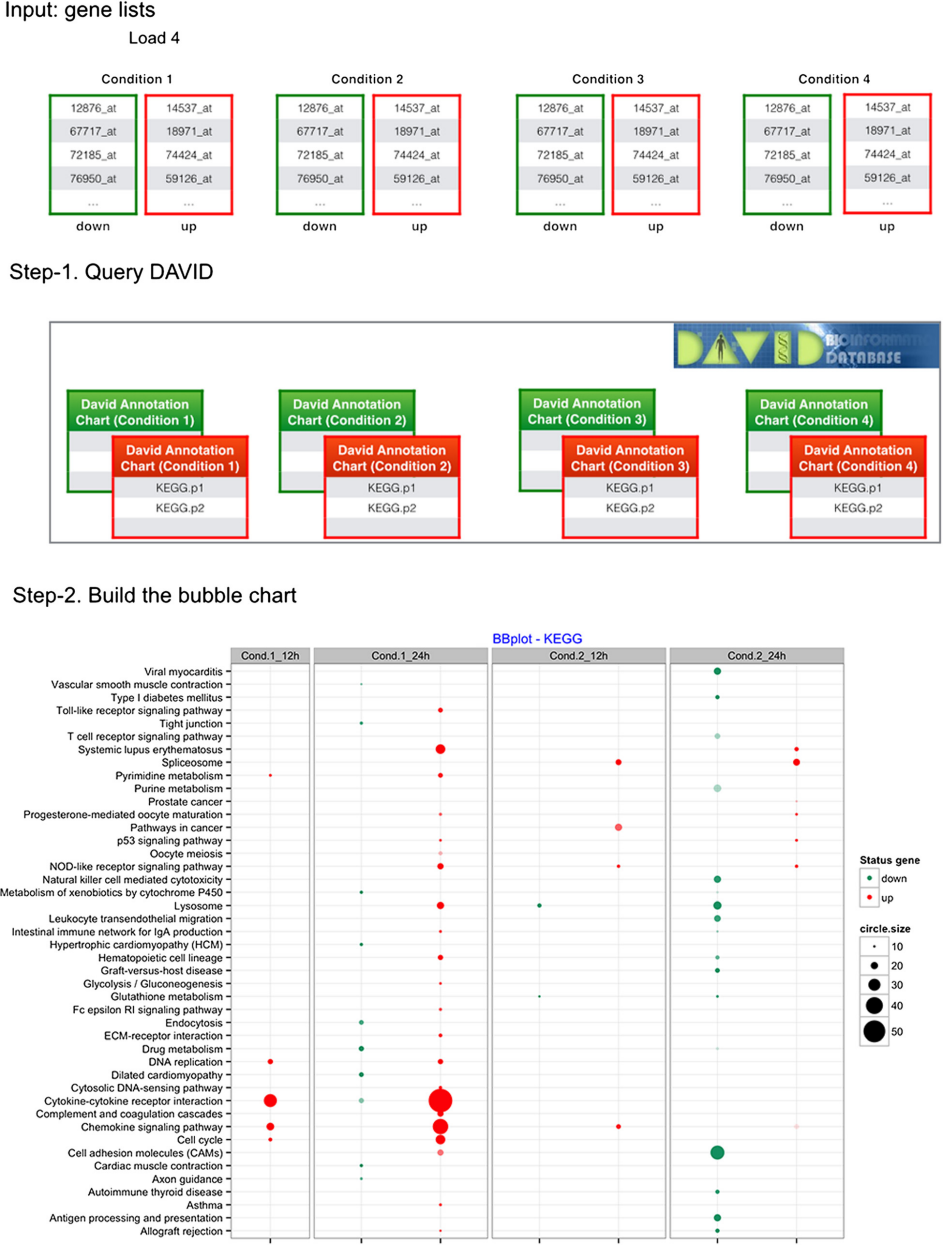
**Main flowchart in BACA.** Input: eight gene lists corresponding to four experimental conditions. Up- and down-regulated genes must be included in two separate lists. Step 1: the DAVIDsearch function loads the eight gene lists, queries the DAVID knowledgebase and generates eight different DAVID annotation charts. The red and green boxes evidence the DAVID charts found with up- and down-regulated genes, respectively. Step 2: the BBplot uses the eight DAVID annotation charts to build a plot comparing DAVID annotations across multiple enrichment results. The chart shows a grid where each row represents an enriched annotation found by DAVID and each column the experimental condition where that annotation was highlighted. While, each cell reports a bubble indicating the number of genes enriching the corresponding annotation and the state of these genes in terms of down- and up-regulation.

BACA has been implemented as package in R. It provides three R functions: DAVIDsearch, BBplot and Jplot. Figure 1 shows the flowchart of the main part of the BACA package. DAVIDsearch is a user-friendly R function that uses the RDAVIDWebService [3] to query DAVID and wrap the results into R objects, namely, DAVIDFunctionalAnnotationChart objects. First, multiple gene lists are uploaded to DAVID, and then an automated enrichment analysis is performed based on a given database/resource (*i.e.*, GO-based terms, KEGG pathways, etc.) for each gene list separately. DAVIDsearch requires registration with DAVID^1^ and other optional input parameters. An important input parameter is the easeScore (or P-value). It can be used to filter the enrichment analysis results. However, we suggest to return all possible annotations (easeScore = 1) and apply a threshold on the significance level when using BBplot. In this way, further queries to DAVID are avoided. DAVIDsearch outputs a list of DAVIDFunctionalAnnotationChart objects, which is used as input of the BBplot function to build a bubble chart like the one shown in Figure 1. This chart displays three dimensions of data. Each entity with its triplet (v_1_, v_2_, v_3_) of associated data is plotted as a bubble that expresses two of the v_i_ values through the disk’s xy location and the third through its size. The disk’s xy location gives the information about an enriched functional annotation (x-axis) associated with a given experimental condition (y-axis). The third dimension, expressed as the size of the bubble, indicates how many genes from a given gene list (y-axis) are associated with an enriched annotation (y-axis). Moreover, the BBplot uses different colors to indicate whether the genes associated with each enriched annotation are down- (default color is green) or up- (default color is red) regulated. The bubble chart in Figure 1 allows the visualization and comparison of the enriched annotations found by using up-/down-regulated gene lists derived from different conditions/experiments.

The BBplot creates a global, synthetic picture showing unique and common functional annotations found by using DAVID. In particular, it shows how common annotations are represented across different experimental conditions. BBPlot function accepts different, optional input parameters. The two most important are p-value (or EASE score) and count. These parameters are useful to select from the results by DAVID the most significant annotations.

**Figure 2:**
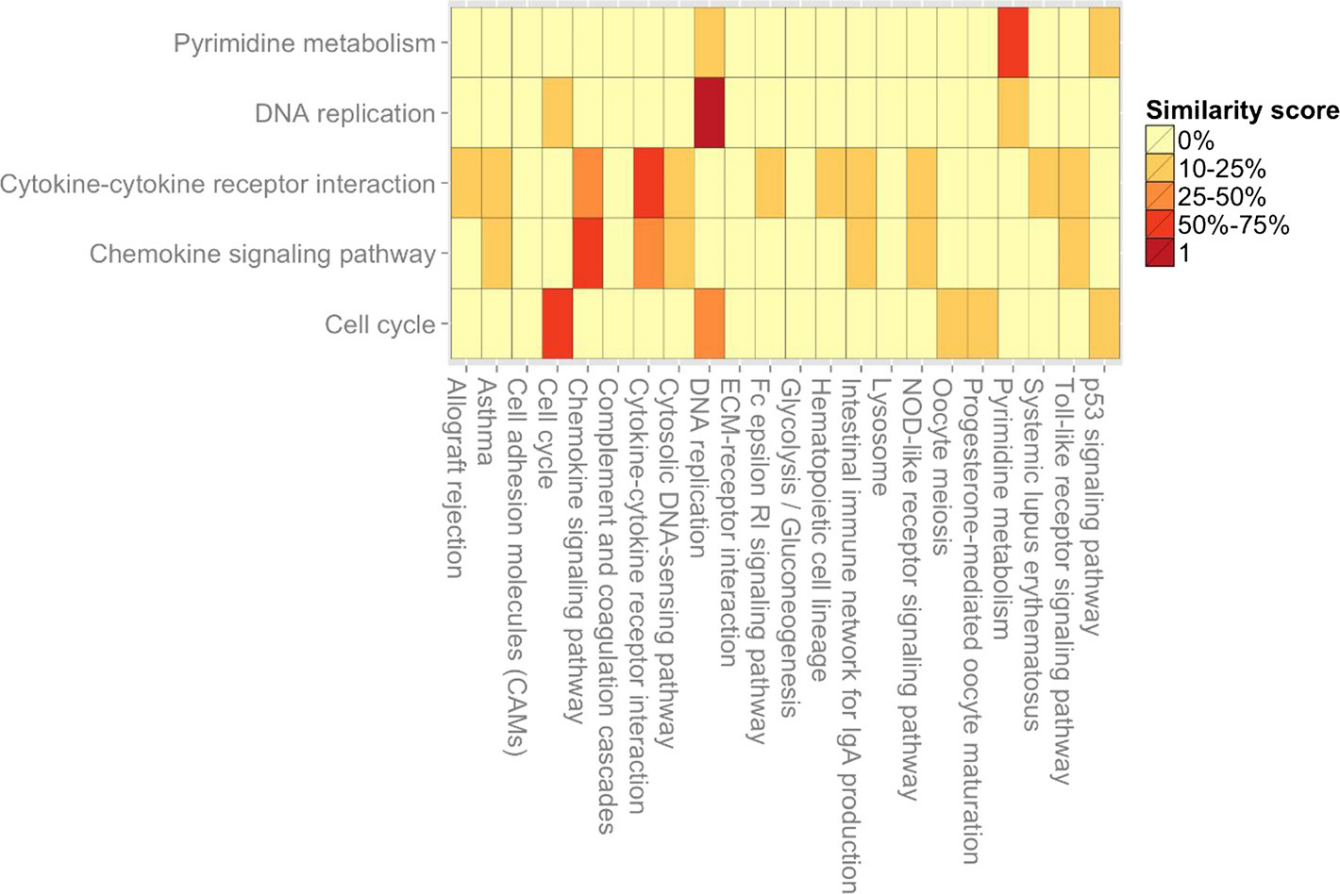
**Example of Jplot.** Input: two DAVID annotation charts objects. The Jplot builds a heatmap showing how similar are the annotations found using different gene lists. The similarity is based on the Jaccard’s coefficient; it is compiled between each pair of annotations x and y, where x and y can be associated, for instance, to two GO-terms or KEGG pathways.

Furthermore, the BACA package provides another graphic function, namely Jplot, to highlight similarities between two enrichment results. Jplot takes in input two DAVIDFunc-tionalAnnotationChart objects and returns a heatmap of Jaccard coefficients showing how similar are the annotations found using different gene lists. The similarity is compiled between the subsets of genes enriching a pair of annotations x and y, where x and y can be associated, for instance, to two GO-terms or KEGG pathway. Figure 2 shows an example of the heatmap built by using Jplot. Additional file 1contains three tables indicating the required/optional input parameters for each developed R-function.

## Results and discussion

BACA is an R package designated to facilitate visualization and comparison of multiple enrichment analysis results. Like any R package, it needs to be installed with all the necessary dependencies. BACA uses external packages and assumes that they are installed. Packages to install and load before to use BACA: RDAVIDWebService [3] and ggplot2 [9]. After installing, the BACA package can be loaded with the command.



In order to carry out quick examples, a set of data is supplied with BACA. This data consists of artificial up- and down-regulated gene lists corresponding to two time points of two different experimental conditions. These gene lists can be loaded with the command.



Once the data is loaded, the R function DAVIDsearch is used to query the DAVID knowledge base.



DAVIDsearch requires two inputs: 1) the lists of up-/down-regulated gene sets and 2) the email of a given registered DAVID users (http://david.abcc.ncifcrf.gov/webservice/register.htm). Additionally, a number of optional parameters can be specified. For instance, the type of submitted ids (*e.g.*, “ENTREZ_GENE_ID”, “GENBANK_ACCESSION”) and the category name (*e.g.*, “GOTERM_BP_ALL”, “KEGG_PATHWAY”, etc.) to be used in the functional annotation analysis can be indicated, as specified in the BACA manual. During the querying process some notes are printed out. They include the name of the gene list, the number of genes successfully loaded, the number of genes mapped (and unmapped) in DAVID, the specie and the number of annotations found by DAVID.



The DAVIDsearch function compiles a list of DAVIDFunctionalAnnotationChart objects, one for each specified gene list. This list is used as input of the *BBplot* function in order to build a chart that shows how the functional annotations found by DAVID have changed across different experimental conditions.



BBplot builds a chart where the annotations are compared by the means of bubbles. The bubble size indicates the number of genes enriching the corresponding annotation, while the colour indicates the state of these genes in terms of down- and up-regulation.

BBplot works out with different optional parameters to filter the enrichment analysis results. In particular, they can use the parameters *max.pval* (or EASE score) and *min.genes* in order to select the most significant enriched annotations. This is necessary when the lists of enriched annotations found by DAVID are very large.

After building the bubble plot, the users can visualize and save it.



Finally, the users can use the Jplot function to build/plot pairwise comparisons between functional annotation charts.



The Jplot function takes in input two different *DAVIDFunctionalAnnotationChart* objects and provides in output a table/matrix with colored boxes. Each box reports the Jaccard index-based similarity score computed between the gene sets enriching two functional annotations.

## Conclusions

The BACA package provides a set of simple R functions to provide visual comparisons of multiple enrichment results obtained by using DAVID.

## Endnotes

^1^http://david.abcc.ncifcrf.gov/webservice/register.htm

## Availability and requirements

BACA is implemented in R and is freely available at the CRAN repository (http://cran.r-project.org/web/packages/BACA/)

- **Project name:** BACA project
- **Project home page:**http://cran.r-project.org/web/packages/BACA/
- **Operating system(s):** Platform independent
- **Programming language:** R
- **Other requirements:** BioC 2.13 (R-3.0)
- **License:** GPL (> = 2)

## Additional file

Additional file 1:
**DOC file including a table of the input arguments for each R function defined in the BACA package.**
